# Tacrolimus-resistant regulatory T cells: Combining the best of both worlds?

**DOI:** 10.1016/j.omta.2026.201805

**Published:** 2026-07-16

**Authors:** Dirk Kuypers

**Affiliations:** 1 Head of Department of Nephrology and Renal Transplantation, University of Leuven, Leuven, Belgium

## Main text

The advancement of adoptive cell therapy has changed perspectives in solid organ transplantation. Immunosuppressive maintenance therapy in transplantation efficiently prevents allograft rejection, but is the main driver of cardio-metabolic complications, death caused by infections, malignancies, and cardiovascular disease.^1^^,^^2^ Minimization strategies for immunosuppression however are not unlimited and not without risk.^3^ Early phase 1/2a studies indicate that adoptive transfer of autologous Tregs in *de novo* living donor kidney allograft recipients is safe and enables reduction of triple-drug immunosuppression to low-dose tacrolimus monotherapy in the majority of patients.^4^^,^^5^^,^^6^^,^^7^ Complete weaning of tacrolimus however is not possible as earlier work demonstrated that regulatory T cells effectively controlled incipient alloreactivity but not memory effector T cells while tacrolimus could do both. Since regulatory T cells strongly depend on IL-2 stimulation for optimal proliferation and function, a combination with tacrolimus is not ideal. An ingenious approach to effectively combine tacrolimus with autologous Tregs without hampering their immunomodulatory function is described in the previous issue of *Molecular Therapy Advances*.^8^

Human FKBP12 knockout Tregs (FKBP12^KO^-Tregs) were developed using a vector-free CRISPR-Cas9 ribonucleoprotein, retaining their functional phenotype, high viability and immunoregulatory capability, comparable to wild-type Tregs (Treg^WT^). FKBP12^KO^-Tregs were impervious to tacrolimus while they remained sensitive to cyclosporin-induced IL-2 inhibition (using another intracellular adaptor protein). This constitutes an excellent fail-safe switch in case FKBP12^KO^-Tregs overactivity or off-target effects would emerge in clinical settings. The authors successfully completed a stringent intricate complex evaluation process and obtained manufacturing authorization for good-manufacturing practice (GMP) processing the FKBP12^KO^-Tregs product (“TregTacRes”). This is the starting point for a first-in-human clinical trial to evaluate safety, dosing and functionality of FKBP12^KO^-Tregs in *de novo* living donor kidney allograft recipients in combination with low-dose tacrolimus.

Gene editing was initiated after achieving high Tregs purity through CD4 enrichment during density gradient centrifugation and flow cytometry sorting, resulting in >90% of CD25^+^FOXP3^+^/CD127^−^CD25^+^ Tregs within CD4^+^ cells with minimum contamination of CD3^−^ and CD3^+^CD8^+^ cells. Gene editing enhanced subsequent cell proliferation stimulated by IL-2 and rapamycin in the presence of anti-CD3/CD28 microbeads. This approach yielded >400 × 10^8^ FKBP12^KO^-Tregs per 50 mL blood, which would be sufficient for clinical dosing of 3 × 10^6^ cells/kg bodyweight, including samples for quality control. Cas9 editing reached 80% efficiency in the final FKBP12^KO^-Tregs product after 21 days expansion. Viable cell recovery and proliferation were actually enhanced in FKBP12^KO^-Tregs compared to Treg^WT^. Expression of FOXP3 in FKBP12^KO^-Tregs was comparable to Treg^WT^ while CD25 expression was (temporarily) lower but with intact STAT5 phosphorylation. The initial yield of unedited Tregs was independent of donor age, which is reassuring when envisioning clinical implementation. Methylation of the Treg-specific demethylation region (TSDR) in FKBP12^KO^-Tregs was <20%, similar to Treg^WT^, and suppression of responder T cell proliferation (Tresp) was FKBP12^KO^-Tregs dose-dependent. Because Tresp proliferation itself is also suppressed by tacrolimus, FKBP12^KO^-Tresp were used instead and again a significantly increased suppressive capacity of FKBP12^KO^-Tregs under tacrolimus treatment could be shown in comparison to Treg^WT^. Addition of everolimus even further increased the suppressive capacity of FKBP12^KO^-Tregs in this setting, which is valuable information when designing a first-in-human clinical trial. Counterintuitively, FKBP12^KO^-Tregs preserve their mTORC1 signaling (even demonstrating increased suppressive capacity) when exposed to everolimus because the latter also depends on FKBP12-binding to execute its growth inhibitory functions through mTOR inhibition. Proteomics indeed confirmed increased LDHA and PGAM1 in FKBP12^KO^-Tregs, necessary for IL-2-dependent PI3K/mTORC1 signaling, FOXP3 expression and Treg homeostasis. Importantly, the proliferative advantage of FKBP12^KO^-Tregs over Treg^WT^ exposed to tacrolimus was not preserved under standard triple immunosuppression (tacrolimus, mycophenolic acid, and corticosteroids). This further indicates that in order to optimize potential clinical benefits of autologous FKBP12^KO^-Tregs co-treatment, dedicated immunosuppressive regimens need to be identified that facilitate FKBP12^KO^-Tregs expansion and function. A better understanding of FKBP12^KO^-Tregs immune regulatory characteristics under tacrolimus treatment (CTLA-4 and PD1 expression ↑; IL-2, TNFR1 and IL-10 signaling ↑; pro-inflammatory cytokines: IFNγ, IL-12/IL-13 and IL-4 signaling ↓) can direct the choice and timing of tailored co-immunosuppression.

Given that high proliferation rates in the context of GMP production could lead to cell exhaustion, *KLRG1*, *TOX*, and *BCL2* gene expression related to reduced longevity, was reassuringly reduced in FKBP12^KO^-Tregs. Protein characterization related to FKBP12^KO^-Tregs proliferation and metabolism showed no signs of increased exhaustion, cellular stress, or dysfunction, which confirms that gene editing did not adversely affect cell function and survival. Transcriptomics confirmed an increased Th2 signature (*IL-5*, *IL-10*, and B cell activation pathways) in FKBP12^KO^-Tregs exposed to tacrolimus compared to Treg^WT^. At the same time, *IL2RA* and *FOXP3* expression remained stable with balanced proliferation marker gene expression for *STAT5A/B*, *MYC*, *SLC2A*, *HEK2*, and *CCND2*. Increased IL-2 signaling in FKBP12^KO^-Tregs under tacrolimus seem to compensate partially for reduced CD25 expression with higher observed gene expression (switch) for *IL2RA* and *IL2RB* instead of *IL2RG*.

This intensive preparatory work lead to effective GMP manufacturing (semi-closed bioreactors) of stable “TregTacRes” products with >94% viable cells of which >95% was CD4^+^CD25^+^FoxP3^+^ including >79% FKBP12^KO^-Tregs. Contamination with CD8^+^ cells and IL-2/IFNγ producers was low. IFNγ produced by alloantigen-reactive Tregs is important for their optimal regulatory function *in vivo* (e.g., context of Th1 inflammation) so the biological relevance of minor proportions of IFNγ producers in the product needs to be further clarified. A yield of 0.65 × 10^6^ Tregs was realized after 18–23 days incubation (>20.000-fold expansion rate) from 50 mL of whole blood, which is a unique result.

After initial screening, reproducibly nominated off-targets across donors occurred predominantly in intronic (45.8%) regions and less in exonic (9.7%) genomic regions. Together with *in silico* nominated sites from reference and populations genomes, a total of 255 putative off-target sites were withheld for off-target interrogation using deep-sequencing. Four sites [*GGA3* (vesicular trafficking), *MRPS7*, *NUP62CL* (nuclear pore-related gene) and *NHS* (rare developmental disease gene)*, ATP11A* (pseudogene)] were identified with significant off-target editing and significant indel frequencies ranging from 0.03% to 0.54%. All were classified as variants of unknown significance (VUS). Interrogation of on-target editing confirmed high frequencies of on-target editing ranging from 97% to 99%. Whether the identified VUS’ in off-target regions have implications will be part of study safety protocols but no relevant impact on Treg biology could be demonstrated. No evidence of clonal expansion, malignant transformation or loss of repertoire diversity was demonstrated in FKBP12^KO^-Tregs.

The field of T-regulatory cell therapy in kidney transplantation is expanding, reassured by results of the pioneering autologous Treg trials showing safe reduction of classic immunosuppressive treatment.^4^^,^^5^^,^^6^^,^^7^ Establishing a stable FKBP12^KO^-Treg product with retained operational immune regulatory functions under tacrolimus exposure, is a timely and exciting addition to the adoptive cell therapeutic repertoire. Another type of autologous Treg modification that could potentially be evaluated in combination with the FKBP12^KO^-Treg phenotype is a recent CAR-Treg product. A first-in-human phase 1/2 dose-escalating study (STEADFAST study) with autologous HLA-A2-restricted CAR-Tregs in HLA-A2-positive living donor transplants was recently concluded (NCT04817774).^9^^,^^10^ Safe reduction of immunosuppression was successful in all actively treated patients and adverse effects were not different from controls.^10^ Furthermore specific immune cell infiltrates, called tolerogenic Treg structures (TOLs) were observed in surveillance renal biopsies at all dose levels.^10^ These focal lymphocytic infiltrates contain prominent CD20^+^ B cell and regulatory signatures (e.g., IL-10, PD-L1, TIGIT), potentially indicating intra-graft T- and B-cell interactions that promote local immune regulation.^11^

It can be envisioned that successful Treg gene editing strategies like demonstrated by the authors, open up enormous possibilities, including development of (off-the-shelf) allogeneic Treg products, optimizing intrinsic Treg survival, metabolism and signaling, and postponing autologous Treg harvesting, editing, and retransferring until after deceased donor transplantation.

## Declaration of interests

D.K. received advisory board fees from Sangamo Therapeutics Inc. (paid to institution).
